# Gastrointestinal Symptoms During Cancer Therapy: The Clinician’s Role

**DOI:** 10.1016/j.advnut.2025.100472

**Published:** 2025-07-11

**Authors:** Robert Dunn

**Affiliations:** Clinical Nutrition Manager, Mass General Cancer Center, Boston, MA, United States

For patients undergoing anticancer therapy, both treatment delay and reduction of intended dose intensity have been associated with decreased overall survival [1,2]. There are many potential causes of treatment interruptions across the cancer continuum; however, a secondary analysis of older adults receiving chemotherapy found that gastrointestinal symptoms were responsible for ∼15% of all unplanned hospitalizations (second only to infection) [3]. An accumulating burden of gastrointestinal symptoms predisposes patients to the “overlap syndromes” of malnutrition, cachexia, and sarcopenia [4]. Declining nutrition status has been recurrently linked to treatment intolerance [5] and lower overall survival [6].

Given the intersectional relationships between symptom burden, malnutrition, and treatment failure, there is an imperative for clinicians to develop approaches to managing these symptoms that are grounded in evidence. In their recent article, Alzoubi and Loman [7] describe the widespread prevalence of gastrointestinal symptoms across various cancer treatment methods. Additionally, they describe limitations of a solely pharmacologic approach to management, reasonably arguing for the importance of medical nutrition therapy in this context.

The authors remark that a “majority” of medical nutrition therapy delivered to patients with cancer is provided “without consideration for the underlying causes of malnutrition.” If true, this would represent a profound failure among oncology nutrition professionals. Thankfully, Alzoubi and Loman’s meta-analysis offers a useful framework for assessing the potential efficacy of various nutrition interventions. Crucially, the article offers situation-specific guidance. Probiotics were found to reduce the incidence of diarrhea in patients being treated with either chemotherapy or radiation, whereas supplementation with omega-3 fatty acids was impactful only in the context of chemotherapy. Additionally, the article’s quantification of effect size (conveniently outlined in Table 3) allows clinicians to stratify interventions by potential impact.

Competent nutrition professionals may integrate single nutrient supplementation where appropriate but should not consider these a panacea. Artificial intelligence tools are rapidly emerging as potential decision-making aides in oncology nutrition [8], and care must be taken to ensure that neither oncology dietitians nor artificial intelligence agents apply a “paint by numbers” approach to nutrition counseling. Patient-specific factors will influence the efficacy of the proposed interventions; probiotics may likely fail to reduce the incidence of diarrhea in patients being treated with chemotherapy with yet-to-be-controlled pancreatic insufficiency.

This review found oral nutrition supplements were not effective tools for managing gastrointestinal symptoms. Still, future products may target specific symptoms by integrating 2 or more nutrients that were found effective in this review – a commercial beverage containing both omega-3 fatty acids and probiotics may be more effective at reducing incidence of diarrhea than currently available products. Additionally, future studies should evaluate whether the concept of food synergy is relevant for this population [9]. Understanding the role of food-first approaches in oncology-specific medical nutrition therapy will empower clinicians to provide more patient-centered guidance.

Of note, the meta-analysis by Alzoubi and Loman [7] excluded articles whose titles included the terms “parenteral nutrition” and “exercise.” Future research efforts should consider the role of parenteral nutrition in patients with symptomatic barriers to adequate oral or enteral nutrition, particularly in populations who may undergo surgical management of their cancer. Additionally, although outside the scope of this review, clinicians should consider the role of exercise interventions in multimodal care models. The American Society of Clinical Oncology has offered guidelines on implementing exercise interventions during cancer treatment, noting their impact on treatment tolerance [10].

Clinicians who provide supportive care to patients during anticancer therapy should, certainly, adopt a holistic approach to nutrition counseling that goes beyond simple macronutrient delivery. Prompt identification and treatment of gastrointestinal symptoms is necessary to avoid the deleterious (and bidirectional) effects of treatment pauses and malnutrition.

## Author contributions

The sole author was responsible for all aspects of this manuscript.

## Funding

The author reports no funding received for this work.

## Conflict of interest

The author reports no conflicts of interest.
